# Association between coffee drinking and telomere length in the Prostate, Lung, Colorectal, and Ovarian Cancer Screening Trial

**DOI:** 10.1371/journal.pone.0226972

**Published:** 2020-01-08

**Authors:** Bella Steiner, Leah M. Ferrucci, Lisa Mirabello, Qing Lan, Wei Hu, Linda M. Liao, Sharon A. Savage, Immaculata De Vivo, Richard B. Hayes, Preetha Rajaraman, Wen-Yi Huang, Neal D. Freedman, Erikka Loftfield

**Affiliations:** 1 Yale School of Public Health, Yale University, New Haven, CT, United States of America; 2 Division of Cancer Epidemiology and Genetics, National Cancer Institute, National Institutes of Health, Bethesda, MD, United States of America; 3 Harvard T.H. Chan School of Public Health, Boston, MA, United States of America; 4 NYU Langone Health, New York, NY, United States of America; University of Hawai'i at Manoa College of Tropical Agriculture and Human Resources, UNITED STATES

## Abstract

Mounting evidence indicates that coffee, a commonly consumed beverage worldwide, is inversely associated with various chronic diseases and overall mortality. Few studies have evaluated the effect of coffee drinking on telomere length, a biomarker of chromosomal integrity, and results have been inconsistent. Understanding this association may provide mechanistic insight into associations of coffee with health. The aim of our study was to test the hypothesis that heavier coffee intake is associated with greater likelihood of having above-median telomere length. We evaluated the cross-sectional association between coffee intake and relative telomere length using data from 1,638 controls from four previously conducted case-control studies nested in the Prostate, Lung, Colorectal, and Ovarian Cancer Screening Trial. Coffee intake was assessed using a food frequency questionnaire, and relative telomere length was measured from buffy-coat, blood, or buccal cells. We used unconditional logistic regression models to generate multivariable-adjusted, study-specific odds ratios for the association between coffee intake and relative telomere length. We then conducted a random-effects meta-analysis to determine summary odds ratios. We found that neither summary continuous (OR = 1.01, 95% CI = 0.99–1.03) nor categorical (OR <3 cups/day vs. none = 1.37, 95% CI = 0.71–2.65; OR ≥3 cups/day vs. none = 1.47, 95% CI = 0.81–2.66) odds ratio estimates of coffee drinking and relative telomere length were statistically significant. However, in the largest of the four contributing studies, moderate (<3 cups/day) and heavy coffee drinkers (≥3 cups/day) were 2.10 times (95% CI = 1.25, 3.54) and 1.93 times as likely (95% CI = 1.17, 3.18) as nondrinkers to have above-median telomere length, respectively. In conclusion, we found no evidence that coffee drinking is associated with telomere length. Thus, it is unlikely that telomere length plays a role in potential coffee-disease associations.

## Introduction

Coffee is one of the most commonly consumed beverages in the world, with a global consumption of 9.8 billion kilograms forecasted for the year June 2018 –June 2019 [1]. In the United States, three out of every four adults drink coffee, with a majority consuming it daily [2]. The 2015–2020 Dietary Guidelines for Americans addressed coffee consumption for the first time, stating that current scientific evidence suggests that three to five 8-oz cups per day can be part of a healthy diet [3]. This conclusion was based on a growing body of literature indicating that moderate coffee consumption does not increase and may even decrease risk of death [4] or age-related chronic diseases, such as type 2 diabetes [5], Parkinson’s disease [6], Alzheimer’s disease [7] and certain cancers, including liver [8], endometrium [9], skin [10] and colon [11]. The potential mechanisms that underlie inverse associations of coffee with certain chronic diseases are not fully understood, but moderate coffee consumption has been inversely associated with inflammatory markers, which may be related to the disease process [12]. Additionally, coffee contains over 1,000 compounds, including bioactive polyphenols, caffeine, and cafestol. These and several other compounds found in the coffee bean have been shown to have antioxidant, antihypertensive, and chemo-preventive properties [13].

There is increasing interest in the relationship between telomere length and lifestyle factors, such as diet, which have also been associated with age-related, chronic diseases. Telomeres are long tandem nucleotide repeats and associated proteins, located at the ends of chromosomes, which maintain chromosomal stability. They shorten with each cell division, serving as markers of cellular aging and replicative ability. Accordingly, shorter telomeres have been associated with greater risk of chronic diseases, such as type 2 diabetes [14], some cancers [15], and ischemic heart disease [16]. In contrast, longer telomeres have been associated with some cancers, such as lung [17], hepatocellular carcinoma in a cohort of Hepatitis B patients [18], and colorectal adenoma [19].

Two recent studies using data from the Nurses’ Health Study (NHS) [20] and the 1999–2002 National Health and Examination Survey (NHANES) [21], found significant associations between higher coffee intake and longer telomere length. Three smaller studies have also looked at this question; one observed a positive association [22] and two were null [23, 24]. Given the inconsistent results to date, we sought to elucidate the relationship by evaluating the cross-sectional association between coffee consumption and relative telomere length. For reasons described herein, we meta-analyzed data of controls from four cancer-related case-control studies nested in the Prostate, Lung, Colorectal, and Ovarian (PLCO) Cancer Screening Trial, a large population-based cohort of U.S. adults with detailed data on dietary intake and other lifestyle factors.

## Methods

### Study population

The PLCO Cancer Screening Trial, which has been described in detail elsewhere [25], commenced in 1993 and enrolled 154,897 men and women, aged 55–74 years at baseline, from 10 screening centers around the United States. The screening phase of the trial included a screening arm, in which participants were screened for cancers of interest at regular intervals, and a usual care arm; following the active trial phase, cohort follow-up continued for mortality and cancer incidence. All-cancer incidence for participants in both trial arms was ascertained via a combination of active follow-up and passive linkage with state cancer registries, which have been detailed elsewhere [26]. All PLCO study participants provided written informed consent before participation, and the study protocols was approved by Institutional Review Boards of each study center and the NCI; and the Yale Human Investigation Committee designated the secondary analysis presented, herein, as “not human subjects research”. We used data from four nested cancer case-control studies that measured relative telomere length (RTL) [17, 27, 28] in the PLCO cohort. Three of these studies were nested within the screening arm, which included blood draws, and the fourth (i.e. glioma) study was nested within the overall PLCO population and used blood or buccal cell DNA. The inclusion criteria, matching factors, and sample sizes for these studies are summarized in S1 Table. Questionnaires administered at baseline were used to ascertain information about diet, demographics, and other lifestyle factors.

**Fig 1:**
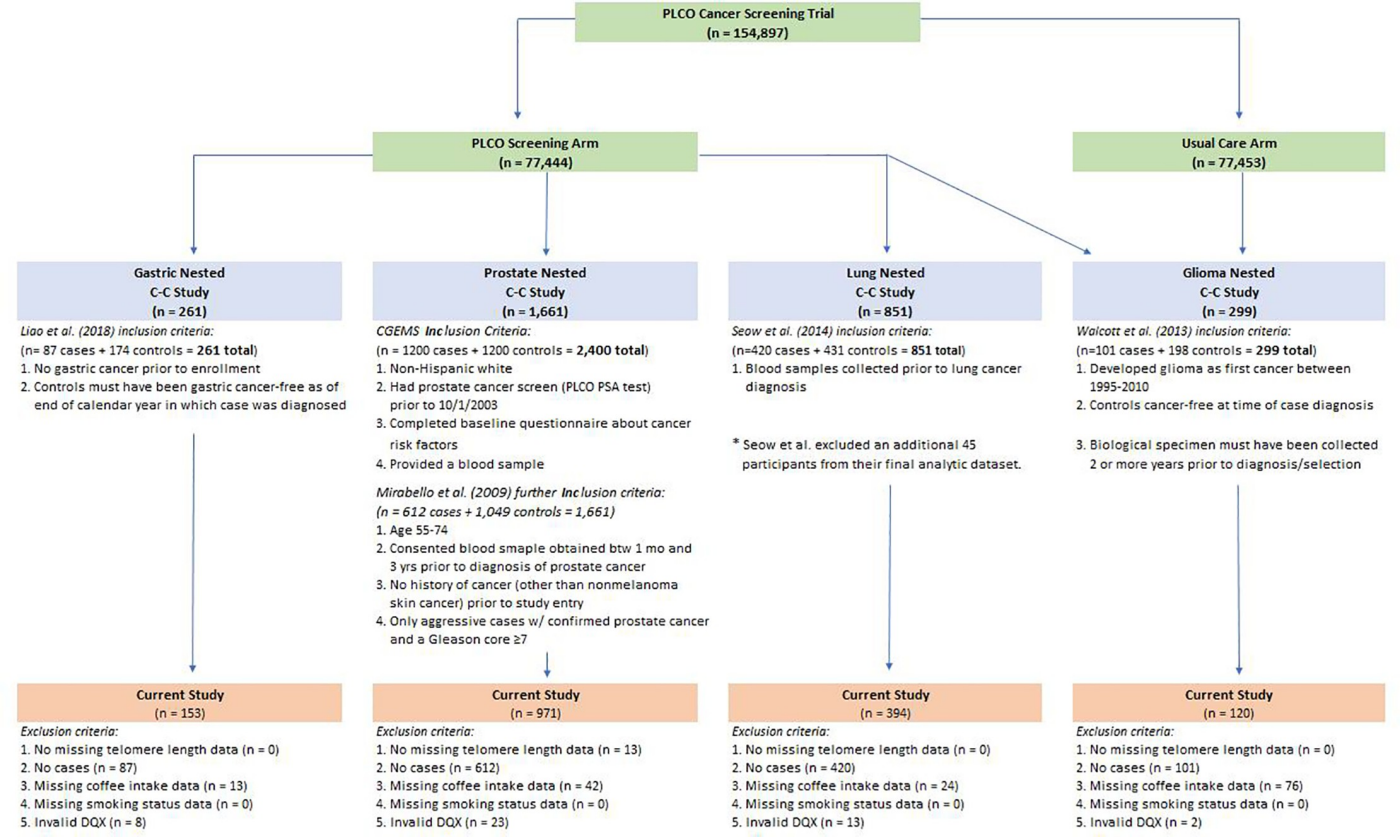
Flowchart illustrating inclusion/exclusion criteria for final analytic sample. Fig 1 shows how we arrived at our final analytic sample size from the PLCO cohort.

There were 261 participants in the gastric cancer study, 1,661 participants in the prostate cancer study, 851 participants in the lung cancer study, and 299 participants in the glioma study. We excluded individuals in the following order: missing RTL (n = 0 gastric, n = 13 prostate, n = 0 lung, n = 0 glioma), cases (n = 87 gastric, n = 612 prostate, n = 420 lung, n = 101 glioma), missing coffee intake (n = 13 gastric, n = 42 prostate, n = 24 lung, n = 76 glioma), and an invalid food frequency questionnaire, known as the dietary questionnaire (DQX) (n = 8 gastric, n = 23 prostate, n = 13 lung, n = 2 glioma). The DQX was considered invalid if a date of completion was not provided, if the participant was not alive when the questionnaire was completed, if eight or more frequency responses were missing, or if reported calorie intake was extreme (first or last percentile by gender). We limited our sample to controls only from each study in order to avoid systematically biasing the effect estimate due to factors stemming from the various cancers of interest and their potential relationship to telomere length or coffee drinking. Our final sample size consisted of 153 gastric cancer controls, 971 prostate cancer controls, 394 lung cancer controls, and 120 glioma controls. Fig 1 illustrates how we arrived at our final analytic sample from the overall PLCO cohort.

### Coffee consumption

Participants self-reported via the DQX whether in the previous 12 months they drank coffee never, less than once per month, 1–3 times per month, 1 time per week, 2–4 times per week, 5–6 times per week, 1 time per day, 2–3 times per day, 4–5 times per day, or 6+ times per day. They also reported their typical serving size (small, medium, or large cup) of coffee.

Grams of coffee intake was estimated by multiplying the reported frequency of consumption and serving size by a gram amount, which depended on the subject’s gender. This gram amount was derived from the USDA’s 1994–1996 Continuing Survey of Food Intakes by Individuals (CSFII) database [29]. We then converted grams to cups using the following conversion: 226.796 grams per 8-ounce cup. Finally, we categorized participants as coffee non-drinkers (0 cups/day), moderate (>0 and <3 cups/day) coffee drinkers, or heavy (≥3 cups/day) coffee drinkers. These categories were used to preserve sample sizes within categories of intake across the four studies.

### Relative telomere length measurements

Telomere length was measured separately for each case-control study by the original investigator. Each of the four nested case-control studies measured telomere length from DNA extracted from either blood, buffy-coat, or buccal cell samples that were acquired at baseline or follow-up prior to study inclusion. DNA was extracted using the Qiagen blood protocol, phenol:chloroform, or ProMega ReliaPrep method from buffy-coat or blood. Telomere length was previously measured during each study’s respective time of data collection using the validated quantitative real-time polymerase chain reaction-based assay (qPCR), as described elsewhere [30, 31]. All assays were performed in triplicate, with blind quality control samples interspersed between test samples to assess inter- and intra-plate variability. Laboratory personnel were blinded to case-control status in the gastric, glioma, and prostate cancer studies.

The RTL variable was calculated as a ratio of telomere repeat copy number to the single-gene copy number (T/S), as compared to a randomly chosen reference group of DNA [17, 27, 28, 30, 31]. Because each case-control study was conducted at different times, used varying approaches to extract DNA, varying DNA sources, and different reference samples for estimating RTL, we elected to conduct a meta-analysis of study-specific measures of association rather than pool the data.

### Statistical analysis

We calculated basic descriptive statistics for each study sample of controls and the overall screening arm in the PLCO population. We then evaluated the association between coffee drinking, RTL, and selected characteristics using chi-square tests for categorical variables and analysis of variance for continuous variables.

We evaluated the association between continuous cups of coffee and study-specific dichotomized RTL using separate unconditional logistic regression models for each study. Logistic regression models were also used to estimate odds ratios (ORs) and 95% confidence intervals (CIs) for the association between categorical coffee intake (< 3 cups/day or ≥ 3 cups/day versus none) and RTL (dichotomized at the study-specific median) within each study separately. We calculated study-specific p-trends using the median value of each coffee intake category in logistic regression models.

All study-specific multivariable models were adjusted for age (years, continuous), sex, cigarette smoking status (never, current, former), years since quitting among former smokers (<10, 10–20, >20 years), number of cigarettes among current or former smokers (≤20 or >20), total daily caloric intake (kcals, continuous), study year of blood draw (continuous), education (college graduate or not), body mass index (BMI) (<25 kg/m2, 25–30 kg/m2, ≥ 30 kg/m2), alcohol consumption (none, <1 drink daily, 1–3 drinks daily, ≥3 drinks daily), physical activity (none, <1 hour, 1–2 hours, ≥ 3 hours per week), daily red and white meat consumption (grams/1,000 kcal), and daily fruit and vegetable consumption (cups/1,000 kcal).

We conducted secondary analyses to explore potential effect modification using the prostate cancer control sample only due to limited sample sizes in the other studies. We assessed interactions with age, BMI, and smoking status (never, current, former) by including the cross-product term for continuous coffee intake and each variable of interest in the multivariable model; statistical significance was evaluated using the Wald chi-square statistic for the interaction variable. All study-specific analyses were conducted using SAS 9.4 software (SAS Institute).

The study-specific multivariable-adjusted ORs were meta-analyzed using a random-effects model to derive summary ORs and 95% CIs. To evaluate study heterogeneity within the random-effects model, we inspected forest plots with corresponding Q and I^2^ statistics; the associated p-values, at a significance level of P≤0.05, determined whether study heterogeneity was present. The meta-analysis was conducted using the metafor package in R [32].

## Results

Table 1 shows the baseline characteristics of the screening arm of the PLCO Cancer Screening Trial and of our control-only samples from the nested case-control studies. The distribution of baseline characteristics was largely similar among the four case-control studies, except for three variables. Median coffee intake was similar for the gastric, prostate, and lung control populations, (3.9 cups/day for the first two and 3.7 cups/day for the latter) but was lower for glioma controls (3.1 cups/day). Three of the control samples were majority male and the prostate sample was entirely male. Race/ethnicity also differed by study since the prostate and glioma studies only included non-Hispanic Whites; 16% and 6% of gastric and lung controls, respectively, reported a race/ethnicity other than non-Hispanic White.

**Table 1:** Baseline characteristics of the PLCO Cancer Screening Trial screening arm and controls in four nested case-control studies within PLCO ^a^.

	PLCO Screening Arm ^b^	Controls from Gastric Cancer Study	Controls from Prostate Cancer Study	Controls from Glioma Study	Controls from Lung Cancer Study
Characteristic	(n = 61376)	(n = 153)	(n = 971)	(n = 120)	(n = 394)
Sex, n (%)					
Female	30530 (49.7)	33 (21.6)	---	43 (35.8)	151 (38.3)
Male	30846 (50.3)	120 (78.4)	971 (100.0)	77 (64.2)	243 (61.7)
Age group, n (%)					
≤59 y	20052 (32.7)	29 (19.0)	196 (20.2)	31 (25.8)	80 (20.3)
60 to 64 y	19166 (31.2)	48 (31.4)	320 (33.0)	38 (31.7)	148 (37.6)
≥65 y	22158 (36.1)	76 (49.7)	455 (46.9)	51 (42.5)	166 (42.1)
Education, n (%)					
Not college graduate	26132 (42.6)	68 (44.4)	371 (38.2)	54 (45.0)	165 (41.9)
College graduate or postgraduate	35191 (57.3)	85 (55.6)	599 (61.7)	66 (55.0)	229 (58.1)
Race/Ethnicity, n (%)					
Non-Hispanic White	55804 (90.9)	128 (83.7)	971 (100.0)	120 (100.0)	371 (94.2)
Other	5554 (9.1)	25 (16.3)	---	---	23 (5.8)
Body mass index, n (%)					
<25 kg/m^2^	20231 (33.0)	50 (32.7)	233 (24.0)	46 (38.3)	119 (30.2)
25 to 30 kg/m^2^	26015 (42.4)	75 (49.0)	499 (51.4)	41 (34.2)	182 (46.2)
≥30 kg/m^2^	14525 (23.7)	26 (17.0)	228 (23.5)	30 (25.0)	90 (22.8)
Smoking status, n (%)					
Never	28767 (46.9)	63 (41.2)	367 (37.8)	62 (51.7)	173 (43.9)
Former	26531 (43.2)	81 (52.9)	501 (51.6)	49 (40.8)	178 (45.2)
Current	6078 (9.9)	9 (5.9)	103 (10.6)	9 (7.5)	43 (10.9)
Alcohol consumption, n (%)					
None	10806 (17.6)	23 (15.0)	136 (14.0)	25 (20.8)	55 (14.0)
<1 drink/day	36411 (59.3)	91 (59.5)	517 (53.2)	70 (58.3)	244 (61.9)
1–3 drinks/day	9206 (15.0)	25 (16.3)	182 (18.7)	15 (12.5)	64 (16.2)
≥3 drinks/day	4953 (8.1)	14 (9.2)	136 (14.0)	10 (8.3)	31 (7.9)
Physical activity, n (%)					
None	9372 (15.3)	25 (16.3)	139 (14.3)	22 (18.3)	66 (16.8)
<1 hour/week	11008 (17.9)	26 (17.0)	164 (16.9)	17 (14.2)	80 (20.3)
1 to 2 hours/week	16986 (27.7)	42 (27.5)	281 (28.9)	33 (27.5)	95 (24.1)
≥3 hours/week	23795 (38.8)	60 (39.2)	385 (39.7)	47 (39.2)	153 (38.8)
Coffee (cups/day), median (IQR)	3.7 (0.7–4.0)	3.9 (0.7–6.9)	3.9 (1.2–6.9)	3.1 (0.2–5.3)	3.7 (1.5–6.7)
Red meat (g/1000 kcal/day), median (IQR)	32.2 (20.2–47.6)	36.2 (23.5–54.2)	38.2 (26.2–55.2)	33.2 (22.7–48.7)	34.8 (22.2–49.1)
White meat (g/1000 kcal/day), median (IQR)	21.3 (13.1–33.4)	20.8 (13.2–35.1)	20.2 (12.4–31.9)	19.6 (11.0–29.1)	20.2 (12.3–32.0)
Fruit (cups/1000 kcal/day), median (IQR)	1.1 (0.7–1.5)	1.0 (0.6–1.4)	0.9 (0.5–1.3)	1.1 (0.8–1.7)	1.0 (0.6–1.5)
Vegetable (cups/1000 kcal/day), median (IQR)	1.3 (1.0–1.6)	1.3 (1.0–1.6)	1.2 (0.9–1.5)	1.2 (1.0–1.5)	1.3 (0.9–1.6)

Abbreviations: Interquartile range (IQR); Prostate, Lung, Colorectal, and Ovarian (PLCO)

^a^ Frequencies and percentages may not sum to total due to missing data and/or rounding

^b^ PLCO screening arm exclusion criteria are invalid DQX, missing coffee intake, and missing cigarette smoking status

In all four studies, higher levels of alcohol drinking and lower levels of fruit consumption were associated with higher coffee consumption (all P<0.05) (S2 Table). Cigarette smoking was strongly associated with heavier coffee drinking in all studies (all P<0.01), except among controls from the glioma study. Longer RTL was associated with younger age in the gastric (P = 0.03), prostate (P<0.0001), and lung (P = 0.003) study control samples, but not in the glioma control sample (Table 2).

**Table 2:** Participant characteristics according to category of RTL ^a^^,^ ^b^.

	CONTROLS FROM GASTRIC CANCER STUDY			CONTROLS FROM PROSTATE CANCER STUDY			CONTROLS FROM GLIOMA STUDY			CONTROLS FROM LUNG CANCER STUDY		
Characteristic	RTL < Median	RTL ≥ Median	P ^c^	RTL< Median	RTL≥ Median	P ^c^	RTL< Median	RTL≥ Median		RTL< Median	RTL≥ Median	P ^c^
	(n = 77)	(n = 76)		(n = 455)	(n = 516)		(n = 60)	(n = 60)	P ^c^	(n = 198)	(n = 196)	
Sex, n (%)			0.88			---	---	---	0.57			0.07
Female	17 (51.5)	16 (48.5)		---	---		23 (53.5)	20 (46.5)		67 (44.4)	84 (55.6)	
Male	60 (50.0)	60 (50.0)		455 (46.9)	516 (53.1)		37 (48.1)	40 (52.0)		131 (53.9)	112 (46.1)	
Age group, n (%)			0.03			<0.001			0.24			0.003
≤59 y	10 (34.5)	19 (65.5)		53 (27.0)	143 (73.0)		13 (41.9)	18 (58.1)		30 (37.5)	50 (62.5)	
60–64 y	21 (43.8)	27 (56.3)		100 (31.3)	220 (68.8)		17 (44.7)	21 (55.3)		69 (46.6)	79 (53.4)	
≥65 y	46 (60.5)	30 (39.5)		302 (66.4)	153 (33.6)		30 (58.8)	21 (41.2)		99 (59.6)	67 (40.4)	
Smoking Status, n (%)			0.56			0.96			0.03			0.01
Never	33 (52.4)	30 (47.6)		172 (46.9)	195 (53.1)		30 (48.4)	32 (51.6)		75 (43.4)	98 (56.7)	
Former	41 (50.6)	40 (49.4)		236 (47.1)	265 (52.9)		29 (59.2)	20 (40.8)		93 (52.3)	85 (47.8)	
Current	3 (33.3)	6 (66.7)		47 (45.6)	56 (54.4)		1 (11.1)	8 (88.9)		30 (69.8)	13 (30.2)	

Abbreviation: Relative telomere length (RTL)

^a^ Frequencies and percentages may not sum to total due to missing data and/or rounding

^b^ Percentages displayed are row %

^c^ P-values for chi-square test statistics

^d^ RTL in lung control sample is log transformed to normalize right-skewed telomere length

**Fig 2:**
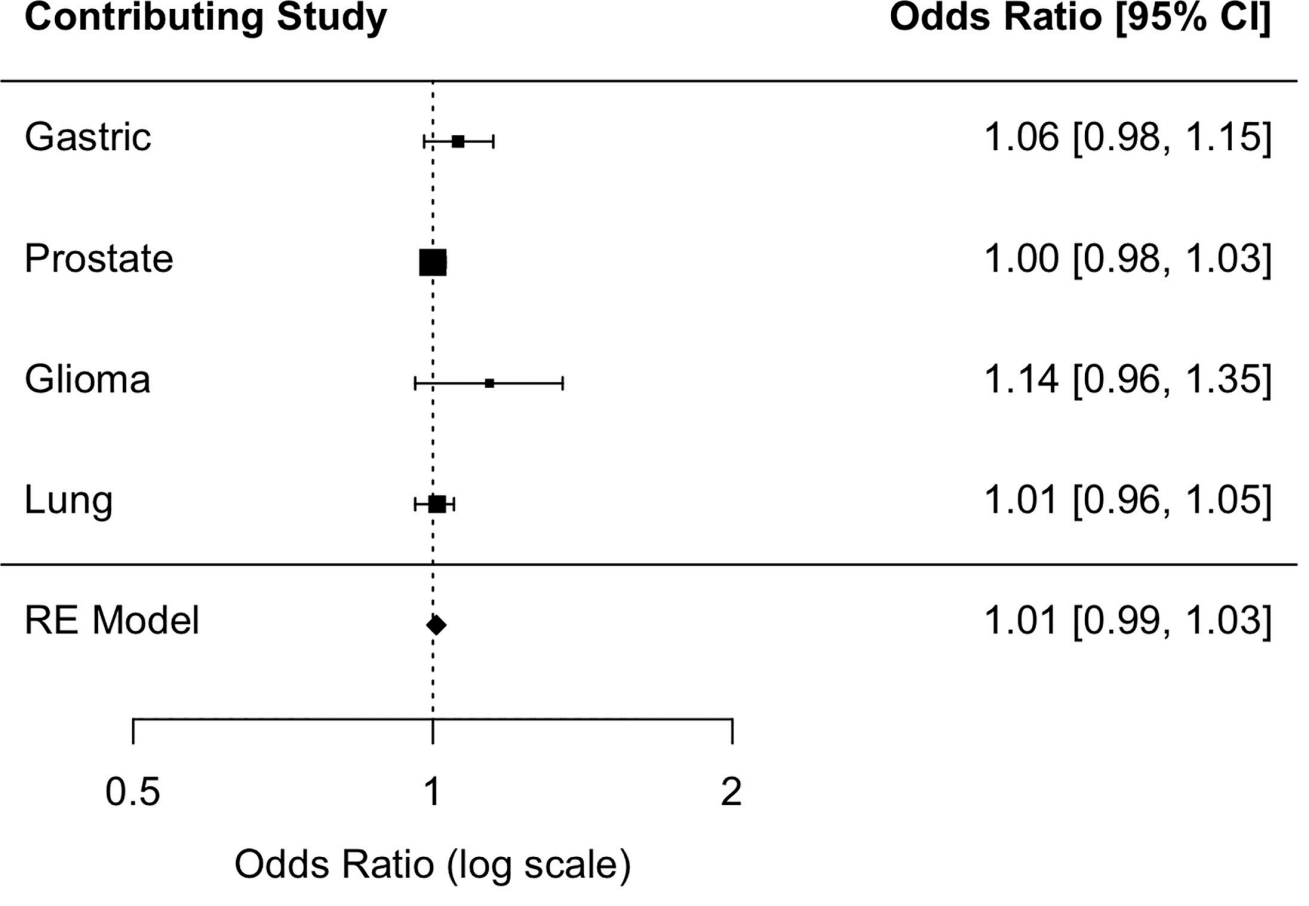
Association between a one-cup increase in coffee consumption and above-median telomere length. Fig 2 shows the individual study odds ratio (OR) estimates and the random-effects (RE) summary OR estimate of the association between a one-cup increase in coffee consumption and above-median relative telomere length among study controls.

**Fig 3:**
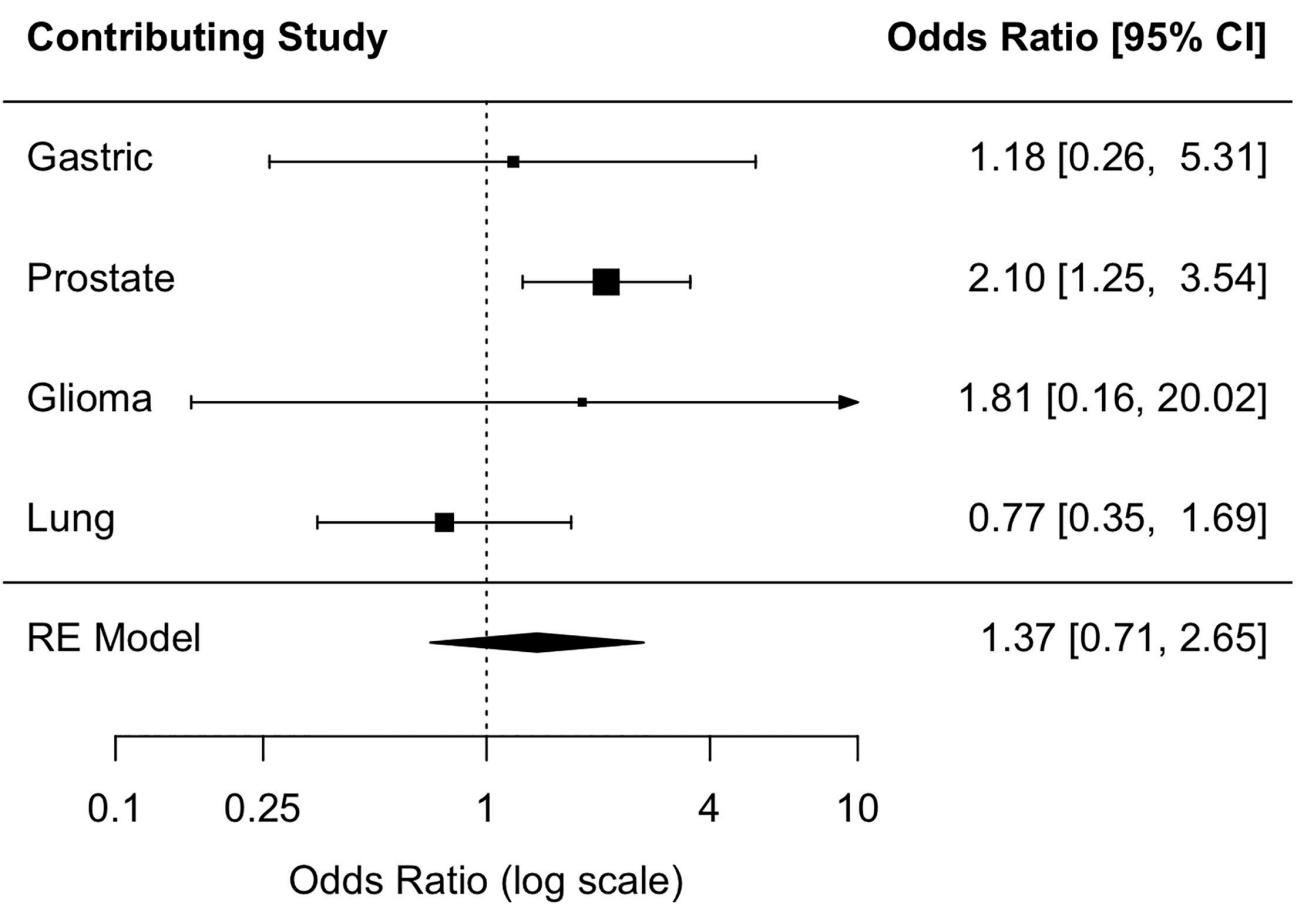
Association between moderate coffee consumption and above-median telomere length. Fig 3 shows the individual study odds ratio (OR) estimates and the random-effects (RE) summary OR estimate of the association between moderate (<3 cups/day) coffee consumption and above-median relative telomere length among study controls.

**Fig 4:**
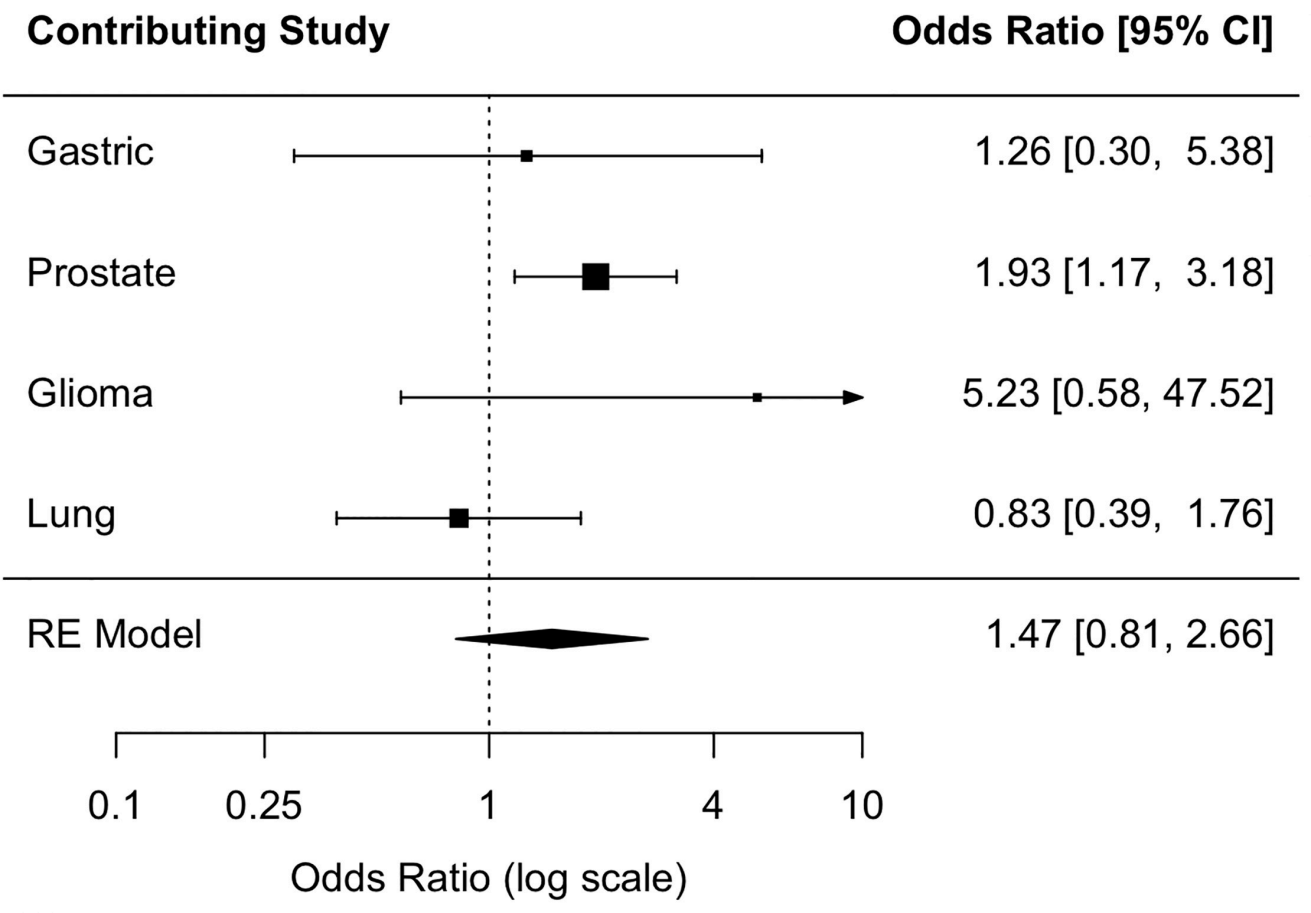
Association between heavy coffee consumption and above-median telomere length. Fig 4 shows the individual study odds ratio (OR) estimates and the random-effects (RE) summary OR estimate of the association between heavy (≥3 cups/day) coffee consumption and above-median relative telomere length among study controls.

The summary OR across the four populations provided no evidence of an association between coffee consumption and RTL (Figs 2, 3 and 4). In the prostate study control sample, which was all male, moderate (<3 cups/day) and heavy coffee drinkers (≥3 cups/day) were 2.10 times (95% CI: 1.25, 3.54) and 1.93 times as likely (95% CI: 1.17, 3.18) as coffee non-drinkers to have above-median RTL, respectively; however, the OR estimate for the continuous coffee intake variable (OR cups/day = 1.00 (0.98, 1.03)) provided no evidence of a linear relationship (Table 3). The direction of the association in the glioma and gastric study control samples, but not the lung control sample, was consistent, albeit not statistically significant, with that of the prostate study control sample. There was no evidence of study heterogeneity (P = 0.27 for continuous one-cup increase, P = 0.21 for moderate coffee drinkers, P = 0.20 for heavy coffee drinkers).

**Table 3:** Odds of long RTL associated with coffee consumption.

		Coffee Consumption				
Sample		Non-Drinkers	Moderate Drinkers (< 3 cups/day)	Heavy Drinkers (≥3 cups/day)	P-trend ^b^	Odds Ratio (1 cup increase/day) ^c^
Controls from Gastric Cancer Study	Coffee (cups/day), median (IQR)	0.0 (0.0–0.0)	0.7 (0.0–1.5)	4.0 (3.9–9.9)		
	< Median RTL, n (%)	8 (53.3)	29 (56.9)	40 (46.0)		
	≥ Median RTL, n (%)	7 (46.7)	22 (43.1)	47 (54.0)		
	OR (95% CI) ^a^	1.00 (ref)	1.18 (0.26, 5.31)	1.26 (0.30, 5.38)	0.79	1.06 (0.98, 1.15)
Controls from Prostate Cancer Study	Coffee (cups/day), median (IQR)	0.0 (0.0–0.0)	1.2 (0.1–1.5)	4.0 (3.9–9.9)		
	< Median RTL, n (%)	55 (58.5)	136 (47.1)	264 (44.9)		
	≥ Median RTL, n (%)	39 (41.5)	153 (52.9)	324 (55.1)		
	OR (95% CI) ^a^	1.00 (ref)	2.10 (1.25, 3.54)	1.93 (1.17, 3.18)	0.25	1.00 (0.98, 1.03)
Controls from Glioma Study	Coffee (cups/day), median (IQR)	0.0 (0.0–0.0)	0.9 (0.1–1.5)	6.7 (3.9–9.3)		
	< Median RTL, n (%)	7 (63.6)	27 (51.9)	26 (45.6)		
	≥ Median RTL, n (%)	4 (36.4)	25 (48.1)	31 (54.4)		
	OR (95% CI) ^a^	1.00 (ref)	1.81 (0.16, 20.02)	5.23 (0.58, 47.52)	0.16	1.14 (0.96, 1.35)
Controls from Lung Cancer Study	Coffee (cups/day), median (IQR)	0.0 (0.0–0.0)	1.5 (0.6–1.5)	4.0 (3.9–9.3)		
	< Median RTL, n (%)	17 (40.5)	66 (52.0)	115 (51.1)		
	≥ Median RTL, n (%)	25 (59.5)	61 (48.0)	110 (48.9)		
	OR (95% CI) ^a^	1.0 (ref)	0.77 (0.35, 1.69)	0.83 (0.39, 1.76)	0.61	1.01 (0.96, 1.05)

Abbreviations: Odds ratio (OR); Confidence interval (CI); Referent group (Ref); Relative telomere length (RTL); Interquartile range (IQR)

^a^ Adjusted for age (year, continuous), sex, cigarette smoking status (never, current, former), years since quitting among former smokers (<10

10–20, >20 years), number of cigarettes among current or former smokers (≤20 or >20), total daily caloric intake (kcals, continuous), study year

of blood draw (continuous), education (college graduate or not), body mass index (<25 kg/m^2^, 25–30 kg/m^2^, ≥ 30 kg/m^2^), alcohol consumption

(none, <1 drink daily, 1–3 drinks daily, ≥3 drinks daily), physical activity (none, <1 hour, 1–2 hours, ≥ 3 hours per week), daily red and white

meat consumption (grams/1,000 kcal), and daily fruit and vegetable consumption (cups/1,000 kcal)

^b^ P-trend calculated using median of each category

^c^ Association between cups of coffee (continuous) and RTL dichotomized at median

Trend tests for the association between coffee drinking and RTL were not statistically significant (Table 3). Additionally, there was no evidence of an association between continuous coffee consumption and dichotomized RTL in any of the individual study control populations (Table 3) or summary ORs (Fig 4). Among prostate cancer controls, we found no evidence of effect modification by age, BMI, or smoking status (S3 Table).

## Discussion

In our meta-analysis of controls from four cancer-related case-control studies nested in the PLCO Cancer Screening Trial, we did not observe a statistically significant association between heavier coffee consumption and above-median RTL. Within our study-specific analyses, there was a positive statistically significant association for moderate and heavy coffee drinkers versus coffee nondrinkers in controls from the largest of the four case-control studies, the prostate control sample only. A prior analysis of data from the prostate cancer study found a statistically significant association between healthier lifestyle scores, defined as low or no cigarette use, higher fruit and vegetable intake, lower BMI, and more physical activity, and longer telomere length [27]. Despite careful adjustment for potential confounders, such as these, in our analysis, residual confounding by unmeasured or poorly measured factors is possible. The relationship between coffee and telomere length in controls from the gastric cancer and glioma studies were not statistically significant; however, effect estimates were consistent in direction with results in the prostate study. We found no evidence of an association between cups of coffee measured as a continuous variable and dichotomized RTL.

Previous studies of the association between coffee and telomere length have yielded inconsistent results. Two recent investigations [20, 21], conducted in demographically-distinct populations, found a statistically significant relationship between higher coffee consumption and longer telomere length. An NHANES analysis included 5,826 racially-diverse men and women, ranging in age from 20 to 84 years [21], whereas a study in NHS included 4,780 white, female nurses, ranging in age from 43 to 69 years [20]. In addition, a small randomized controlled trial reported a positive relationship between greater coffee intake and longer telomere length [22]. In this cross-over trial, 37 hepatitis C patients were randomly assigned to drink 4 cups of coffee per day or to abstain from coffee drinking for 30 days; after 30 days, participants switched groups. The researchers found telomere length was significantly higher in participants during the period of coffee drinking than the period of abstention [22]. However, two other studies, both cross-sectional in design, did not find statistically significant associations [23, 24] between coffee drinking and telomere length, but statistical power to detect a modest association may have been an issue.

Limitations of our study include the cross-sectional nature of our analysis, which cannot demonstrate temporality. Although we utilized a food frequency questionnaire (FFQ) that was based on two previously validated FFQs [33, 34] to ascertain coffee intake, FFQ responses generally tend to underestimate true dietary consumption, and resulting measurement error may bias effect estimates. However, prior literature on the validity of self-reported coffee consumption mitigates this concern. Longitudinal studies of regularly-consumed foods, like coffee, have found responses to a semi-quantitative FFQ to be reliable and reproducible [35, 36]. To increase statistical power, we included all available telomere length data measured in cancer-free controls in the PLCO study; nevertheless, we estimate that we had approximately 80% power to detect a modest association of 1.53 but lacked sufficient power to detect a weaker association. The glioma study included RTL measurements on blood and buccal cell DNA; however, this dataset contributed the least number of observations thus excluding these estimates from the meta-analysis did not meaningfully impact our null findings. Moreover, the original glioma case-control study from which this data was sourced found no difference in results based on sample source [28]. In addition, we were unable to pool the raw data from each of the four studies because our relative measure of telomere length could not be standardized across studies. Another limitation is the possibility of residual confounding by unmeasured or poorly measured factors that are correlated with coffee drinking and causally related to telomere length. Finally, our dietary dataset did not distinguish between caffeinated and decaffeinated coffee consumption, so we could not evaluate the association between caffeine and telomere length, which may be relevant based on the results of prior studies [20, 21].

The present investigation has several strengths. One is our granular categorization of cigarette smoking status, in which beyond characterizing non-, former, and current smokers, we also adjusted our models by years since quitting (for former smokers) and number of cigarettes smoked per day (for former and current smokers). Coffee and cigarette smoking are highly correlated [4, 37, 38] and our detailed characterization reduces the possibility of residual confounding by smoking status. Also, with the exception of the prostate cancer study control sample, the underlying studies in our analysis included a mix of men and women, contributing to the generalizability of our overall summary estimates. Additionally, we maximized our statistical power by conducting a meta-analysis of the study-specific results. The validated coffee consumption data within the PLCO cohort is another strength; self-reported data for participants who completed two food frequency questionnaires, three years apart, had a Spearman correlation coefficient of 0.76 [12]. The rich data of the PLCO trial allowed us to comprehensively adjust for possible confounders. Moreover, because each of the studies was drawn from the same cohort, we were able to adjust for confounders consistently across each study. Finally, all participants were cancer-free throughout follow-up, minimizing the potential effect of preclinical disease on baseline telomere length or coffee intake.

## Conclusions

We did not find evidence for an association between coffee drinking and telomere length in the PLCO cohort. Thus, it is unlikely that telomere length plays a role in observed coffee-disease associations. Nevertheless, we cannot exclude the possibility of a modest association. Experimental studies as well as epidemiologic studies with still larger sample sizes and diverse populations may further clarify the association between coffee drinking and telomere length.

## Supporting information

S1 Table Study characteristics of four cancer-related nested case-control studies in the PLCO cancer screening trial. (DOCX)

S2 Table Participant characteristics according to level of coffee intake. (DOCX)

S3 Table Stratified odds of long RTL associated with coffee consumption in the prostate control sample. (DOCX)
