# Supplementary material for: Association between coffee drinking and telomere length in the Prostate, Lung, Colorectal, and Ovarian Cancer Screening Trial
Source: PLoS One. 2020 Jan 8;15(1):e0226972. doi: 10.1371/journal.pone.0226972 (PMC6948744; doi:10.1371/journal.pone.0226972)
Supplement: S1 Table — (DOCX) [file pone.0226972.s001.docx]

| **S1 Table.** Study Characteristics of Four Cancer-Related Nested Case-Control Studies in the PLCO Cancer Screening Trial | | | | |
| --- | --- | --- | --- | --- |
| **Author, Year (ref.)** | **Cancer** | **Sample Size** | **Inclusion Criteria** | **Matching Factors** |
| Unpublished data | Gastric | 87 cases; 174 controls | Cases and controls were those without a prior gastric cancer, sampled from the PLCO screening arm. Controls must have been gastric cancer-free as of the end of the calendar year in which the case was diagnosed. | Age level (≤59, 60-64, 65-69, 70+); race (White, Black, Hispanic, Asian, Pacific Islander); gender; year of randomization |
| Mirabello et al., 2009 | Prostate | 612 cases; 1049 controls | Mirabello et al. sampled from CGEMS: Cases and controls were men from the PCLO screening arm of non-Hispanic white race/ethnicity; completed baseline questionnaire about cancer risk factors; provided blood sample; and for cases had prostate cancer screen prior to October 1, 2003.  Additional criteria for Mirabello et al.: ages 55-74 years; no history of cancer (other than non-melanoma skin cancer) prior to study entry; and for cases consented blood sample obtained between 1 month and 3 years prior to diagnosis of prostate cancer; confirmed prostate cancer and Gleason score of ≥7. | Age at cohort entry (5-year intervals); year since initial screen (1-year time window); fiscal year of cohort entry ^a^ |
| Walcott et al., 2013 | Glioma | 101 cases; 198 controls | Cases were men and women who developed glioma as a first cancer between 1995-2010.  Controls were cancer-free at time of case diagnosis.  Biological specimen must have been collected 2 or more years prior to diagnosis/selection. | Trial randomization arm (control arm or intervention arm); gender; age at baseline in 4 categories (55-59, 60-64, 65-69, 70+); calendar year of randomization; cigarette smoking status (never, current, former); calendar year of blood draw; fully matched on DNA source ^b^ |
| Seow et al., 2014 | Lung | 403 cases; 403 controls | Blood samples collected prior to lung cancer diagnosis | Age at baseline; sex; race; PLCO study center; data of baseline blood draw |
| Abbreviations: Prostate, Lung, Colorectal, and Ovarian (PLCO), Cancer Genetic Markers of Susceptibility (CGEMS)  ^a^ Matching factors pertain to the CGEMS study  ^b^ 17 cases were matched with only one control | | | | |
