# Supplementary material for: Association between coffee drinking and telomere length in the Prostate, Lung, Colorectal, and Ovarian Cancer Screening Trial
Source: PLoS One. 2020 Jan 8;15(1):e0226972. doi: 10.1371/journal.pone.0226972 (PMC6948744; doi:10.1371/journal.pone.0226972)
Supplement: S2 Table — (DOCX) [file pone.0226972.s002.docx]

| \| **S2 Table.** Participant Characteristics According to Level of Coffee Intake ^a, b^ \| \| --- \| | | | | | | | | | | | | | | | | |
| --- | --- | --- | --- | --- | --- | --- | --- | --- | --- | --- | --- | --- | --- | --- | --- | --- | --- |
|  | **CONTROLS FROM**  **GASTRIC CANCER STUDY** | | | | **CONTROLS FROM**  **PROSTATE CANCER STUDY** | | | | **CONTROLS FROM**  **GLIOMA STUDY** | | | | **CONTROLS FROM**  **LUNG CANCER STUDY** | | | |
|  | **Coffee Consumption** | | |  | **Coffee Consumption** | | |  | **Coffee Consumption** | | |  | **Coffee Consumption** | | |  |
| **Characteristic** | **None** | **< 3 c/day** | **≥3 c/day** | **P ^c^** | **None** | **< 3 c/day** | **≥3 c/day** | **P ^c^** | **None** | **< 3 c/day** | **≥3 c/day** | **P ^c^** | **None** | **< 3 c/day** | **≥3 c/day** | **P ^c^** |
|  | (n=15) | (n=51) | (n=87) |  | (n=94) | (n=289) | (n=588) |  | (n=11) | (n=52) | (n=57) |  | (n=42) | (n=127) | (n=225) |  |
| Sex, n (%) |  |  |  | 0.27 |  |  |  | --- |  |  |  | 0.78 |  |  |  | 0.89 |
| Female | 5 (15.2) | 13 (39.4) | 15 (45.5) |  | --- | --- | --- |  | 5 (11.6) | 18 (41.9) | 20 (46.5) |  | 17 (11.3) | 50 (33.1) | 84 (55.6) |  |
| Male | 10 (8.3) | 38 (31.7) | 72 (60.0) |  | 94 (9.7) | 289 (29.8) | 588 (60.6) |  | 6 (7.8) | 34 (44.2) | 37 (48.1) |  | 25 (10.3) | 77 (31.7) | 141 (58.0) |  |
| Age group, n (%) |  |  |  | 0.02 |  |  |  | 0.12 |  |  |  | 0.21 |  |  |  | 0.54 |
| ≤59 y | 7 (24.1) | 5 (17.2) | 17 (58.6) |  | 20 (10.2) | 51 (26.0) | 125 (63.8) |  | 6 (19.4) | 11 (35.5) | 14 (45.2) |  | 7 (8.8) | 22 (27.5) | 51 (63.8) |  |
| 60 to 64 y | 4 (8.3) | 15 (31.3) | 29 (60.4) |  | 30 (9.4) | 84 (26.3) | 206 (64.4) |  | 2 (5.3) | 16 (42.1) | 20 (52.6) |  | 18 (12.2) | 45 (30.4) | 85 (57.4) |  |
| ≥65 y | 4 (5.3) | 31 (40.8) | 41 (54.0) |  | 44 (9.7) | 154 (33.9) | 257 (56.5) |  | 3 (5.9) | 25 (49.0) | 23 (45.1) |  | 17 (10.2) | 60 (36.1) | 89 (53.6) |  |
| Education, n (%) |  |  |  | 0.87 |  |  |  | 0.54 |  |  |  | 0.12 |  |  |  | 0.19 |
| Not college graduate | 6 (8.8) | 24 (35.3) | 38 (55.9) |  | 29 (7.8) | 111 (29.9) | 231 (62.3) |  | 2 (3.7) | 27 (50.0) | 25 (46.3) |  | 18 (10.9) | 45 (27.3) | 102 (61.8) |  |
| College grad/ postgrad | 9 (10.6) | 27 (31.8) | 49 (57.7) |  | 65 (10.9) | 178 (29.7) | 356 (59.4) |  | 9 (13.6) | 25 (37.9) | 32 (48.5) |  | 24 (10.5) | 82 (35.8) | 123 (53.7) |  |
| Race/Ethnicity, n (%) |  |  |  | 0.06 |  |  |  | --- |  |  |  | --- |  |  |  | <0.0001 |
| Non-Hispanic White | 12 (9.4) | 38 (29.7) | 78 (60.9) |  | 94 (9.7) | 289 (29.8) | 588 (60.6) |  | 11 (9.2) | 52 (43.3) | 57 (47.5) |  | 37 (10.0) | 112 (30.2) | 222 (59.8) |  |
| Other | 3 (12.0) | 13 (52.0) | 9 (36.0) |  | --- | --- | --- |  | --- | --- | --- |  | 5 (21.7) | 15 (65.2) | 3 (13.0) |  |
| Body mass index, n (%) |  |  |  | 0.82 |  |  |  | 0.82 |  |  |  | 0.09 |  |  |  | 0.50 |
| <25 kg/m^2^ | 6 (12.0) | 19 (38.0) | 25 (50.0) |  | 25 (10.7) | 76 (32.6) | 132 (56.7) |  | 2 (4.4) | 22 (47.8) | 22 (47.8) |  | 10 (8.4) | 46 (38.7) | 63 (52.9) |  |
| 25 to 30 kg/m^2^ | 6 (8.0) | 25 (33.3) | 44 (58.7) |  | 45 (9.0) | 140 (28.1) | 314 (62.9) |  | 7 (17.1) | 12 (29.3) | 22 (53.7) |  | 24 (13.2) | 51 (28.0) | 107 (58.8) |  |
| ≥30 kg/m^2^ | 3 (11.5) | 6 (23.1) | 17 (65.4) |  | 23 (10.1) | 70 (30.7) | 135 (59.2) |  | 2 (6.7) | 15 (50.0) | 13 (43.3) |  | 8 (8.9) | 29 (32.2) | 53 (58.9) |  |
| Smoking status, n (%) |  |  |  | <0.01 |  |  |  | <0.0001 |  |  |  | 0.18 |  |  |  | <0.001 |
| Never | 13 (20.6) | 21 (33.3) | 29 (46.0) |  | 60 (16.4) | 142 (38.7) | 165 (45.0) |  | 7 (11.3) | 32 (51.6) | 23 (37.1) |  | 29 (16.8) | 62 (35.8) | 82 (47.4) |  |
| Former | 2 (2.5) | 29 (35.8) | 50 (61.7) |  | 31 (6.2) | 130 (26.0) | 340 (67.9) |  | 3 (6.1) | 18 (36.7) | 28 (57.1) |  | 12 (6.7) | 57 (32.0) | 109 (61.2) |  |
| Current | 0 (0.0) | 1 (11.1) | 8 (88.9) |  | 3 (2.9) | 17 (16.5) | 83 (80.6) |  | 1 (11.1) | 2 (22.2) | 6 (66.7) |  | 1 (2.3) | 8 (18.6) | 34 (79.1) |  |
| Alc. consumption, n (%) |  |  |  | <0.01 |  |  |  | <0.0001 |  |  |  | 0.04 |  |  |  | 0.08 |
| None | 5 (21.7) | 5 (21.7) | 13 (56.5) |  | 41 (30.2) | 32 (23.5) | 63 (46.3) |  | 6 (24.0) | 9 (36.0) | 10 (40.0) |  | 11 (20.0) | 17 (30.9) | 27 (49.1) |  |
| <1 drink/day | 10 (11.0) | 36 (39.6) | 45 (49.5) |  | 40 (7.7) | 179 (34.6) | 298 (57.6) |  | 3 (4.3) | 36 (51.4) | 31 (44.3) |  | 26 (10.7) | 83 (34.0) | 135 (55.3) |  |
| 1-3 drinks/day | 0 (0.0) | 3 (12.0) | 22 (88.0) |  | 6 (3.3) | 41 (22.5) | 135 (74.2) |  | 1 (6.7) | 5 (33.3) | 9 (60.0) |  | 4 (6.3) | 20 (31.3) | 40 (62.5) |  |
| ≥3 drinks/day | 0 (0.0) | 7 (50.0) | 7 (50.0) |  | 7 (5.2) | 37 (27.2) | 92 (67.7) |  | 1 (10.0) | 2 (20.0) | 7 (70.0) |  | 1 (3.2) | 7 (22.6) | 23 (74.2) |  |
| Physical activity, n (%) |  |  |  | 0.93 |  |  |  | 0.28 |  |  |  | 0.21 |  |  |  | 0.92 |
| None | 2 (8.0) | 8 (32.0) | 15 (60.0) |  | 12 (8.6) | 39 (28.1) | 88 (63.3) |  | 4 (18.2) | 6 (27.3) | 12 (54.6) |  | 5 (7.6) | 20 (30.3) | 41 (62.1) |  |
| <1 hour/week | 4 (15.4) | 9 (34.6) | 13 (50.0) |  | 17 (10.4) | 51 (31.1) | 96 (58.5) |  | 0 (0.0) | 7 (41.2) | 10 (58.8) |  | 8 (10.0) | 24 (30.0) | 48 (60.0) |  |
| 1 to 2 hours/week | 3 (7.1) | 13 (31.0) | 26 (61.9) |  | 20 (7.1) | 89 (31.7) | 172 (61.2) |  | 5 (15.2) | 13 (39.4) | 15 (45.5) |  | 10 (10.5) | 32 (33.7) | 53 (55.8) |  |
| ≥3 hours/week | 6 (10.0) | 21 (35.0) | 33 (55.0) |  | 45 (11.7) | 108 (28.1) | 232 (60.3) |  | 2 (4.3) | 25 (53.2) | 20 (42.6) |  | 19 (12.4) | 51 (33.3) | 83 (54.3) |  |
| Red meat (g/1000 kcal/day), median (IQR) | 29.8  (19.9-46.1) | 32.9  (19.2-42.8) | 38.9  (26.2-61.0) | 0.17 | 33.9  (23.7-50.6) | 37.1  (23.8-53.9) | 38.9  (27.7-55.6) | 0.32 | 29.2  (17.9-39.4) | 32.8  (21.8-55.2) | 34.4  (23.5-47.1) | 0.34 | 32.0  (24.8-41.5) | 32.8  (18.3-43.9) | 35.5  (25.4-54.7) | 0.004 |
| White meat (g/1000 kcal/day), median (IQR) | 20.3  (8.0-38.6) | 22.5  (14.9-36.8) | 20.3  (12.7-31.8) | 0.78 | 22.6  (14.0-39.6) | 20.0  (12.4-31.9) | 20.1  (12.3-31.2) | 0.25 | 18.2  (7.3-31.4) | 19.0  (10.1-27.2) | 20.2  (11.3-30.1) | 0.58 | 21.9  (12.1-30.7) | 23.7  (12.7-38.0) | 19.2  (12.3-28.1) | 0.02 |
| Fruit (cups/1000 kcal/day), median (IQR) | 1.4  (0.7-1.7) | 1.2  (0.7-1.7) | 0.9  (0.6-1.2) | 0.02 | 0.9  (0.6-1.4) | 0.9  (0.6-1.3) | 0.9  (0.5-1.3) | 0.03 | 1.4  (0.6-1.8) | 1.4  (0.8-1.9) | 1.0  (0.7-1.4) | 0.04 | 1.3  (0.8-2.3) | 1.3  (0.7-1.7) | 0.9  (0.5-1.3) | <0.0001 |
| Vegetable (cups/1000 kcal/day), median (IQR) | 1.2  (0.9-1.5) | 1.3  (1.0-1.7) | 1.3  (1.0-1.6) | 0.64 | 1.2  (0.9-1.4) | 1.2  (0.9-1.5) | 1.2  (0.9-1.5) | 0.24 | 1.2  (0.8-1.3) | 1.3  (1.0-1.6) | 1.2  (0.9-1.5) | 0.35 | 1.3  (0.8-1.6) | 1.4  (1.1-1.7) | 1.2  (0.9-1.5) | 0.02 |
| Abbreviation: Interquartile range (IQR); Alcohol (Alc.); Graduate (grad); Cups (c); P-value (P)  ^a^ Frequencies and percentages may not sum to total due to missing data and/or rounding  ^b^ Percentages displayed are row %  ^c^ P-values for categorical and continuous variables correspond to chi-square and analysis of variance (ANOVA) test statistics, respectively | | | | | | | | | | | | | | | | |
