# Supplementary material for: Association between coffee drinking and telomere length in the Prostate, Lung, Colorectal, and Ovarian Cancer Screening Trial
Source: PLoS One. 2020 Jan 8;15(1):e0226972. doi: 10.1371/journal.pone.0226972 (PMC6948744; doi:10.1371/journal.pone.0226972)
Supplement: S3 Table — (DOCX) [file pone.0226972.s003.docx]

| **S3** **Table.** Stratified Odds of Long RTL Associated with Coffee Consumption in the Prostate Control Sample ^a^ | | | | | | |
| --- | --- | --- | --- | --- | --- | --- |
| **Characteristic** | RTL< Median | RTL≥ Median | **Non-**  **drinkers** | **Moderate Coffee Drinkers (< 3 cups/day)** | **Heavy Coffee**  **Drinkers (≥3 cups/day)** | P for  heterogeneity |
|  | n (%) | n (%) | OR (95% CI) ^b^ | OR (95% CI) ^b^ | OR (95% CI) ^b^ |  |
| Age group |  |  |  |  |  | 0.91 |
| ≤59 | 53 (27.0) | 143 (73.0) | 1.00 (ref) | 1.92 (0.46, 7.96) | 1.51 (0.42, 5.42) |  |
| 60 to 64 | 100 (31.3) | 220 (68.8) | 1.00 (ref) | 2.42 (0.98, 5.97) | 2.52 (1.08, 5.89) |  |
| ≥65 | 302 (66.4) | 153 (33.6) | 1.00 (ref) | 2.41 (1.03, 5.65) | 2.18 (0.95, 5.04) |  |
| Body mass index |  |  |  |  |  | 0.36 |
| <25 kg/m^2^ | 122 (52.4) | 111 (47.6) | 1.00 (ref) | 1.04 (0.38, 2.87) | 1.45 (0.56, 3.78) |  |
| 25 to 30 kg/m^2^ | 225 (45.1) | 274 (54.9) | 1.00 (ref) | 2.92 (1.34, 6.35) | 2.99 (1.42, 6.28) |  |
| ≥30 kg/m^2^ | 104 (45.6) | 124 (54.4) | 1.00 (ref) | 2.19 (0.68, 7.07) | 1.18 (0.39, 3.61) |  |
| Smoking Status |  |  |  |  |  | 0.69 |
| Never | 172 (46.9) | 195 (53.1) | 1.00 (ref) | 1.72 (0.86, 3.43) | 1.63 (0.82, 3.22) |  |
| Former | 236 (47.1) | 265 (52.9) | 1.00 (ref) | 3.09 (1.25, 7.67) | 2.83 (1.20, 6.66) |  |
| Current | 47 (45.6) | 56 (54.4) | 1.00 (ref) | 0.31 (0.01, 7.02) | 0.47 (0.03, 8.48) |  |
| Abbreviations: Odds ratio (OR); Confidence interval (CI); Referent group (Ref); Relative telomere length (RTL)  ^a^ Frequencies and percentages may not sum to total due to missing data and/or rounding  ^b^ Adjusted for age (year, continuous), sex, cigarette smoking status (never, current, former), years since quitting among former smokers (<10, 10-20, >20 years), number of cigarettes among current or former smokers (≤20 or >20), total daily caloric intake (kcals, continuous), study year of blood draw (continuous), education (college graduate or not), body mass index (<25 kg/m2, 25-30 kg/m2, ≥ 30 kg/m2), alcohol consumption (none, <1 drink daily, 1-3 drinks daily, ≥3 drinks daily), physical activity (none, <1 hour, 1-2 hours, ≥ 3 hours per week), daily red and white meat consumption (grams/1,000 kcal), and daily fruit and vegetable consumption (cups/1,000 kcal) | | | | | | |
